# Quality Grading of River Crabs Based on Machine Vision and GA-BPNN

**DOI:** 10.3390/s23115317

**Published:** 2023-06-03

**Authors:** Han Wang, Hong Zhu, Lishuai Bi, Wenjie Xu, Ning Song, Zhiqiang Zhou, Lanying Ding, Maohua Xiao

**Affiliations:** 1 College of Engineering, Nanjing Agricultural University, No. 40 Dianjiangtai Road, Pukou District, Nanjing 210031, China; 9203011819@stu.njau.edu.cn (H.W.); 9203012001@stu.njau.edu.cn (L.B.); dinglying@njau.edu.cn (L.D.); 2 Jiangsu Agricultural Machinery Development and Application Center, Nanjing 210017, China; jsnjzhuhong@126.com; 3 College of Economics and Management, Nanjing Agricultural University, Xiaoling Wei Street Weigang No.1, Xuanwu District, Nanjing 210095, China; 4 Kunshan Agricultural Machinery Promotion Station, Kunshan 215300, China

**Keywords:** machine vision, feature extraction, BP neural network, genetic algorithm, data training

## Abstract

The prices of different quality river crabs on the market can vary several times. Therefore, the internal quality identification and accurate sorting of crabs are particularly important for improving the economic benefits of the industry. Using existing sorting methods by labor and weight to meet the urgent needs of mechanization and intelligence in the crab breeding industry is difficult. Therefore, this paper proposes an improved BP neural network model based on a genetic algorithm, which can grade the crab quality. We comprehensively considered the four characteristics of crabs as the input variables of the model, namely gender, fatness, weight, and shell color of crabs, among which gender, fatness, and shell color were obtained by image processing technology, whereas weight is obtained using a load cell. First, mature machine vision technology is used to preprocess the images of the crab’s abdomen and back, and then feature information is extracted from the images. Next, genetic and backpropagation algorithms are combined to establish a quality grading model for crab, and data training is conducted on the model to obtain the optimal threshold and weight values. Analysis of experimental results reveals that the average classification accuracy reaches 92.7%, which proves that this method can achieve efficient and accurate classification and sorting of crabs, successfully addressing market demand.

## 1. Introduction

Crab is an important aquatic product because its delectable taste is widely loved by people; simultaneously, crab also contains proteins, vitamins, and various microelements needed by the human body to prevent diseases, demonstrating an excellent effect. In recent years, the scale of the crab industry has been broadening with the expansion of sales channels and the increase in demand. Crab is basically sold as fresh product; thus, its sales price is deeply affected by the speed, quality, and freshness of the market. However, the quality judgment and sorting of river crabs are mainly conducted manually in the current market. The crab is weighed manually and then judged by observing the shape of its belly umbilicus. However, the shell and claws of crabs are hard and have prickly feet, causing many difficulties in manual grading. Simultaneously, manual sorting is easily affected by various subjective factors, resulting in low sorting efficiency and difficulty in meeting market listing speed, freshness, and seasonal needs. The adoption of manual sorting has also caused enormous cost expenditure, which is not conducive to the stimulation of the vitality of the industry and restricts the far-reaching development of the crab breeding industry. By contrast, automation technology started late in China with a low level of development, and the crab industry in foreign countries is relatively cold. Therefore, studies on the precision automatic sorting of crabs in the world are lacking. Some existing machine sorting devices must manually put the crab onto a conveyor belt. The crab is weighed using the weighing scale on the conveyor belt and then grouped to the designated work area by the sorting device through the lever. Regardless of important factors that affect the quality of river crabs, such as gender, weight, and the color of the crab shell, this device only relies on weight for sorting. This type of machine only has a single sorting index, resulting in low classification accuracy. When judging the quality of river crabs, choosing crabs with turquoise green shells that have a sufficient luster and rich crab meat is still the best option. Combined with this standard, mature machine vision technology and feature extraction method are used to develop an improved BP neural network classification model based on genetic algorithm (GA), which can complete the multi-index quality classification task of crabs and promote the integration of aquaculture and mechanical automation development.

Machine vision [1] with automation, intelligence, high precision, and other characteristics can replace the role of human eyes and is widely used in transportation, automobile manufacturing, visual navigation, defect detection, textile processing, agriculture, and other fields. Compared with other detection methods, one of the advantages of machine vision technology is that it does not need to make direct contact with the detected object, avoids object damage, and improves the accuracy of the work. Machine vision has also been extensively utilized in the field of quality discrimination. Lu et al. [2] used machine vision technology and watershed segmentation method to obtain the white stripe characteristics of *Portunus trituberculatus* and studied the stripes on its shell. Qiu et al. [3] used machine vision technology to grade beef quality by extracting beef marbling using an improved fuzzy C-means clustering algorithm. Chawgien et al. [4] employed acoustic signal and image processing techniques to classify watermelon sweetness using eight ML models. Blasco et al. [5] utilized machine vision technology to construct an online sorting system for apples, oranges, and peaches using Bayesian algorithms for discrimination. However, the above research mainly applies machine learning methods, which have poor generalization capability, whereas neural networks have strong robustness and memory and nonlinear mapping capabilities. Wu et al. [6] established an artificial neural network model for assessing the quality of drinking water. Liu [7] introduced typical applications of various artificial neural networks in fault diagnosis. Among them, BP neural networks have been gradually developed and improved and have been used by many scholars. Luo et al. [8] used BP neural networks to predict the surface roughness of laser-selective melting-formed parts. Shao et al. [9] established a BP-ANN model for soil quality assessment in arid regions of the northwest. Training a suitable network model is usually impossible due to the inability of BP neural networks to stand out from the local optimal solution. Some researchers used GAs, particle swarm optimization, and summation autoregressive moving average models to optimize BP to improve the accuracy and precision of the model and address the aforementioned problem. Chen et al. [10] established an MIV-POS-BPNN network model with BP neural network as the core, the average impulse value method (MIV) as the data preprocessing method, and particle swarm optimization (PSO) as the key parameter for optimization. Yu et al. [11] introduced chaos theory and krill colony algorithm into BP neural networks and established a BP neural network optimization prediction model (C-KHA-BP) for chaotic krill colony algorithm with weights and thresholds as variables to address the minimum error of training. Xu et al. [12] used GA and PSO to improve the generalization capability of BPNN and combined PSO and GA to establish a PSO-GA-BPNN model.

**Figure 1:**
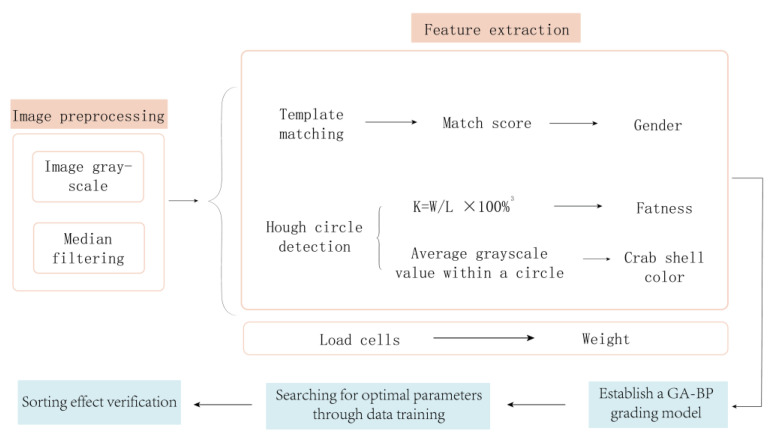
Overall technical route.

Based on the above status quo, this article aims to address the problems of low efficiency, low intelligence, and poor market response in crab sorting operations. In close combination with machine vision theory, an image processing intelligent technology is used to design a method for distinguishing male and female crabs, fat and thin crabs, and crab shell color. Using BP neural networks optimized by GAs, a crab quality grading model based on weight, gender, fatness, and color is established. This model reduces the error rate of manual grading and achieves efficient transmission from the origin to the market. Moreover, the proposed model aims to break through multiple key technologies, such as male and female discrimination, quality identification, and precise fat and thin sorting of Chinese mitten crab, laying a technical foundation for the wide application of intelligent sorting and grading of agricultural products, especially live aquaculture products. The technical route adopted in this paper is shown in Figure 1.

The rest of this article is organized as follows. Section 2 mainly introduces the image preprocessing technology applied to crab images. Section 3 explains the feature extraction techniques of crabs in the following three aspects: gender, fatness, and crab shell color. Section 4 describes BP neural network optimization using GA to establish a crab quality grading model. Section 5 discusses performance verification experiments on the GA-BPNN model. Section 6 summarizes this study.

## 2. Image Preprocessing

Image collection is easily hindered by the environment. Simultaneously, a considerable amount of useless information exists in the images for crab sorting. Image preprocessing is often necessary to remove useless information and provide reliable support for the subsequent quality grading process, which helps achieve good performance in the early stage of quality sorting. This experiment adopts image preprocessing methods of image graying and filtering according to the needs of crab image processing.

### 2.1. Grayscale Processing

**Figure 2:**
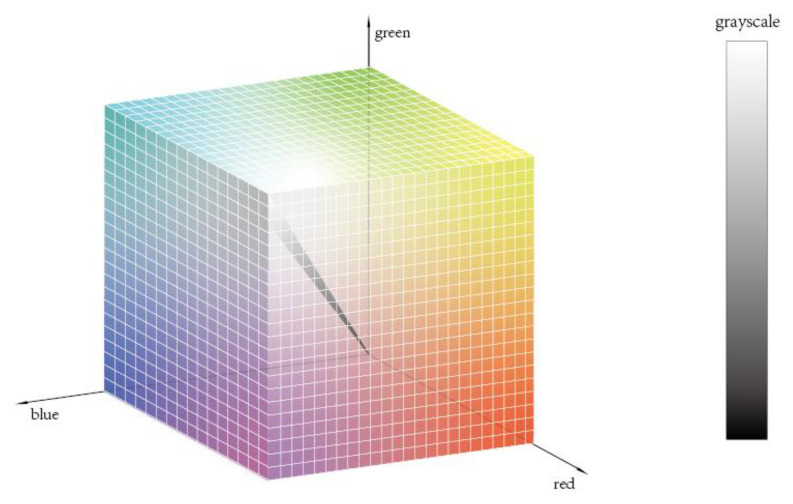
Image color and grayscale representation.

The image output by the camera is a color image, and the image display is based on the “RGB” mode, comprising three colors: red, green, and blue. Any color can be represented by a set of RGB values. The three colors can be mixed in different proportions to represent 2563 colors. Meanwhile, the grayscale image has no color and can only represent the brightness values within the range of 0 (black) to 255 (white). The line from the origin to the maximum value of the RGB model is the grayscale representation. The two modes are shown in Figure 2.

**Figure 3:**
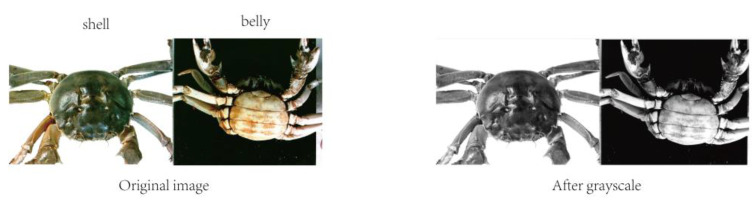
Image grayscale processing.

The color image contains a considerable amount of information. Therefore, this image accounts for a substantial amount of storage space and requires considerable computation, whereas the grayscale image only shows image brightness characteristics. The information contained in the grayscale image is sufficient for river crab quality inspection. Thus, converting the color image into a grayscale one can substantially increase the speed of the entire image processing approach and meet the efficiency requirements for quality inspection. When the pixel coordinates *R* = *G* = *B*, the color image can be converted into a grayscale image, requiring only one byte per pixel for the storage of the grayscale values, which saves a significant amount of space [13]. The common methods for grayscaling color images are component, maximum, average, and weighted average. Among these methods, the weighted average approach weighs the three components according to their importance, resulting in a clearer and more reasonable image than the three other methods. The human eye is most and least sensitive to green and blue, respectively. Thus, the following equation is used to weigh the RGB components for this method to maximize the simulation of the human eye and obtain a reasonable grayscale image [14]:

(1) Y=0.299R+0.587G+0.114B

where *Y* denotes the gray value of the image, and the result of crab image graying is shown in Figure 3.

### 2.2. Image Noise Reduction

Image noise reduction, also known as image filtering, refers to the elimination of noise mixed with the image in the channel transmission while retaining the detailed features of the image. The filtered image is substantially clear and can provide good conditions for subsequent feature extraction. A noisy image can be expressed in a mathematical expression as [15]:

(2) f(x,y)=g(x,y)+η(x,y)

where f(x,y), g(x,y), and η(x,y) are the gray value functions of the noise-added image, the original image, and the noise, respectively.

Three commonly used spatial domain filtering algorithms are as follows: mean, Gaussian, and median filtering [16]. Mean filtering involves providing a template to a target pixel on the image that includes surrounding neighboring pixels and then replacing the original pixel value with the average of all the pixels in the template. This type of filtering ineffectively protects the image details and destroys them while denoising them, resulting in blurred images. Gaussian filtering is a low-pass filter that effectively filters out random Gaussian noise in the image but smooths the edge information in the image while eliminating the noise signal in the image, resulting in a blurred image as well. Median filtering is a nonlinear signal processing method that mainly uses the logical relationship between the original image and the template to obtain results and overcomes the blurring of image details caused by common linear filters under certain conditions. As common noise, pepper noise is generally manifested as black and white pixels in bright or dark areas, respectively. Median filtering is also effective in overcoming pretzel noise; this filtering type can be used to protect edge information and has a simple and fast algorithm. Therefore, the median filtering method is used for noise reduction.

The basic principle of median filtering is to replace the gray value of a pixel with the median of the gray value of the neighborhood of that pixel, allowing the surrounding pixel values to be close to the true value, which eliminates isolated noise points [17]. The median filter handles continuous image window functions similar to a linear filter. However, the filtering process is no longer a weighting operation. Considering pixel processing, the size of the filter kernel is first selected. The pixel values in the neighborhood are then sorted from smallest to largest. Finally, the median value is selected to replace the pixel value at the original center point. The grayscale function of the image after median filtering to remove the noise is expressed in the following formula.

(3) g(x,y)=med{f(x−k,y−l),(k,l∈W)}

In the formula, *f*(*x*, *y*), *g*(*x*, *y*) are the original image and the processed image, respectively, and W is a two-dimensional template, usually in the 3 × 3, 5 × 5 regions, or different shapes.

**Figure 4:**
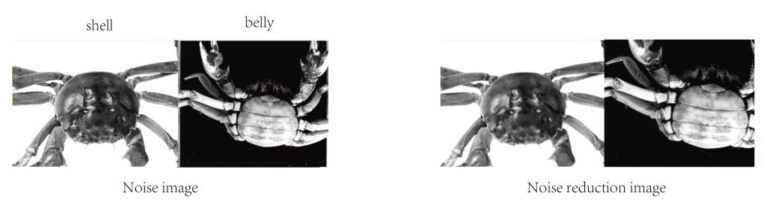
Image noise reduction processing.

The image of the crab after median filtering is shown in Figure 4, which reveals the absence of blurring and damage to important information, such as edges and contours. Simultaneously, the reflective areas on the back of the crab due to the light source are also substantially dark after the median filtering process and the visual effect is optimized. Thus, the purpose of image filtering is achieved.

## 3. Crab Feature Extraction

Feature extraction aims to extract key and useful feature information from a substantial amount of image information. Therefore, the obtained feature vectors can effectively describe the target. Identification of the quality of crabs primarily focuses on several features, such as gender, fatness, and crab shell color.

### 3.1. Gender Feature Extraction

The commonly used image feature extraction methods currently include texture, color extraction, and shape feature extraction. Texture features mainly describe homogeneous phenomena in images; color features are used to describe the surface characteristics of the target; shape features are divided into contour and region shapes according to the different extracted regions [18].

**Figure 5:**
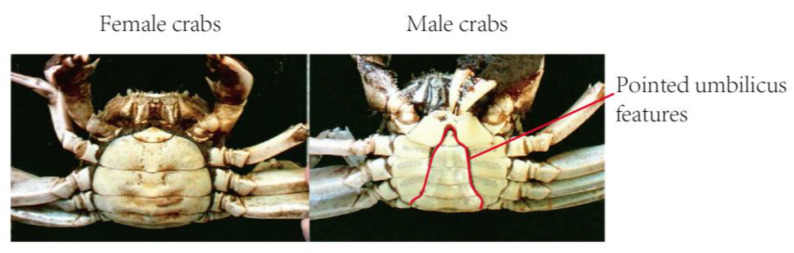
Abdominal umbilical features of male and female crabs.

As shown in Figure 5, the fastest way to distinguish between male and female crabs is to observe the shape of the umbilicus on their abdomen: sharp and round umbilicus indicate male and female crabs, respectively. First, the shape features of the belly button of a crab are extracted using machine vision to distinguish between males and females. Then, template matching is employed to achieve target classification. Template matching can quickly locate predefined targets in the test image by comparing the template and test images to find similar parts on the test image to the template image. Predefined targets can be quickly located in the test image by calculating the similarity between the template image and the targets in the test image. The main principle is as follows: use a target prototype, create a template based on such a prototype, search the test image for the most similar target to the template image, and find the region closest to the average or variance of the template. The rotation angle, row and column coordinates, and zoom size of the target image can be obtained through template matching, achieving feature search on the target image and performing similarity matching. Moreover, template matching has been widely used in defect detection. Qi Xuping [19] employed multiple template matching to achieve surface quality detection of glass containers. Poonam Poonia et al. [20] performed accurate recognition of palm print using template matching. This method demonstrates good robustness and anti-interference performance, realizing the expected matching results for partially occluded situations. Bundling of crabs will not affect the final female crab discrimination. Therefore, template matching is chosen to distinguish between males and females.

When creating a matching template, selecting a region of interest (RIO) and using the pointed navel of the male crab as the template image T is necessary to match the shape of the crab’s abdomen to allow the shape in image I to be detected. The template slides from left to right and from top to bottom on the original image to find similarities between the two. The template can be rotated and scaled during the matching process to meet the needs of crab activities. A metric is calculated at each (*x*, *y*) position to indicate whether the match is good or bad. This experiment uses the correlation matching method to calculate the similarity score between template T and the base image I. The calculation process is simple, and the matching accuracy is high. The calculation formula is as follows:

(4) $$R(x,y)=\sum_{x^{\prime },y^{\prime }}\left(T(x^{\prime },y^{\prime })\cdot I(x+x^{\prime },y+y^{\prime })\right).$$

After the matching is completed, a matching score ranging from 0 to 1 will be output. A large number indicates a high degree of matching. To improve matching accuracy, a score larger than or equal to 0.7 indicates that the crab in the image is male and that lower than 0.7 indicates that the crab in the image is female.

### 3.2. Fatness Feature Extraction

A fat crab body generally indicates superior quality. Therefore, a fatness standard must be introduced to achieve crab quality grading. The formula used to calculate the fatness is:

(5) $$K=\frac{W}{L^{3}}\times 100\%$$

where W is the body weight (g) and L is the length of the crab shell (cm).

The crab weight is obtained using a weighing sensor, and the resistance strain gauge can record and transmit the pressure changes to the host computer to obtain the crab weight. The Hough transform method is used to obtain the carapace length.

Hough transform is a feature extraction method widely used in image analysis and machine vision. This technology can calculate the local maximum of cumulative results in a parameter space to obtain a set that conforms to the specific shape due to Hough transform. The classical Hough transform can only be used to detect straight lines in images. This method was later extended to the recognition of arbitrarily shaped objects, mostly circles and ellipses. Hough line transform is a transform used to detect straight lines. The advantage of the Hough line transform lies in its detection of complete lines in the face of dashed lines or lines that are partially missing or occluded. Hoff circle transform is a machine vision technology used to detect circular objects in an image. The main function of this technology is to track the intersection points between the curves corresponding to each point in the image. If the number of curves intersecting at a point exceeds the set threshold value, then the parameter represented by this intersection point can be considered a circle in the original image. Hoff circle detection is widely used in digital image recognition. Liu Jing et al. [21] located the position of the circular meter from the Hoff circle detection results. Mao Qingzhou et al. [22] used iterative Hough circle transformation to achieve reliable counting of bundled bars. Observation results revealed that the crab shell can be approximately seen as a circle, and the necessary information regarding the length of the shell can be regarded as the diameter of the circle [23]. Hoff circle detection not only detects circular objects in images but also calculates their position and size, which provides convenience for the calculation of the carapace length. Before the Hoff circle transformation, performing the first part of the image preprocessing steps is necessary to reduce noise in the image; otherwise, it may cause serious distortion in Hoff circle detection. The standard Hough circle transformation uses three parameters, namely the center coordinates (*x*, *y*) and the radius r, to accumulate parameters in three-dimensional space to extract a circle. The center coordinates and radius of the circle in image space are determined by establishing a cone cluster in three-dimensional space. However, the calculation amount in three-dimensional space is substantially large, and application of the standard Hough circle transformation is difficult in practice. OpenCV provides a flexible detection method, namely the Hough gradient method, which mainly aims to find the center of a circle based on the modulus vectors that must be located at each point on the circumference. The intersection of these modulus vectors is the position of the circle center, where the modulus vector refers to the vertical line of the tangent line of the point on the circumference.

**Figure 6:**
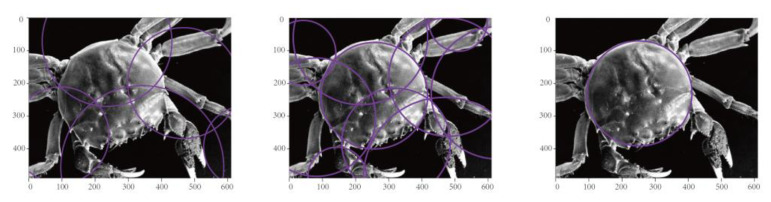
Inspection for crab shell Hoff circle.

Using the Hough gradient method focusing on the radius and the center coordinates of the circle, the circle’s center location was first obtained after two rounds of filtering. The 3D cumulative plane was transformed into a 2D cumulative plane, and the radius size was then determined on the basis of the degree of support from nonzero pixels on the edges of all candidate centers. The choice of some parameters, such as the minimum distance between circle centers (minDist) and the number of votes that must be received at the circle center position (param2), must be considered when running the program. As shown in Figure 6, an excessive number of circles are detected when the minimum distance between centers and the number of votes that must be received at the center position is too small, which can affect the determination of crab shell length. Otherwise, the circle where the crab shell is located cannot be detected and length information cannot be obtained. Therefore, correctly adjusting the parameters, obtaining a suitable Hoff circle detection map, and accurately determining the length information of the carapace are necessary. Notably, the radius measured by a Hoff circle is generally a pixel size. Conversion of the pixel size into actual size involves the following processes. The camera position must be fixed in advance, and a sample crab must be placed with a known carapace length on the conveyor belt as a reference. Photos are then taken to obtain the pixel size, the ratio of its actual size to the pixel size is calculated, and the ratio is utilized for subsequent Hough circle detection to obtain the actual size based on the pixel size.

### 3.3. Extraction of Crab Shell Color Characteristics

Crabs with dark crab shells and a cyan-black appearance under light sources are generally believed to have superior quality. The grayscale processing of the image shows that the grayscale value of the image pixel can be calculated using Formula (1). The radius and center information of the Hoff circle detected in the fatness feature acquisition are used to create a template. While removing other information interference, the average value of the gray value in the template area is calculated and the average value is used as the gray value of the crab shell. A high gray value denotes high crab shell brightness.

## 4. Establishment of Crab Grading Model

After image processing and feature extraction, the information on gender, fatness, and crab shell color can be obtained and then combined with the weight information of crabs to build the crab quality grading model.

### 4.1. BP Neural Network Hierarchical Model

**Figure 7:**
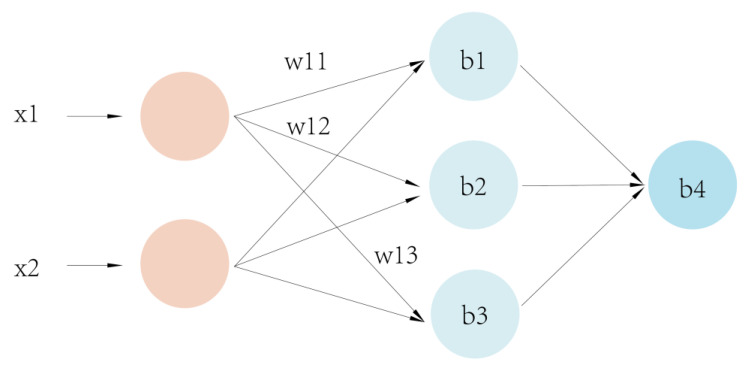
BP neural network infrastructure.

Model classification methods mainly include statistical pattern recognition, structural pattern recognition, neural networks, and deep learning [24]. Among these methods, neural networks can imitate animal neural networks by linking multiple neurons together according to certain rules to form a network, which is divided into input, intermediate, and output layers and is able to achieve nonlinear fitting. The BP neural network model (back-propagation algorithm) is an important artificial neural network branch first proposed by Rumelhart et al. [25]. This model can adjust the weights and thresholds of the network during data training based on feedforward neural networks [26] and achieve nonlinear mapping of the input and output to approximate any nonlinear continuous function with a small error [27]. The underlying structure of this model is shown in Figure 7. BP neural networks not only have a strong nonlinear mapping capability but also possess strong self-learning and self-adaptive capabilities. These networks can automatically extract the “reasonable rules” between input and output data through learning and adaptively remember the learning content in the network weights. Even if the system is locally damaged, it can still work normally and is especially suitable for solving problems with complex internal mechanisms. This network has a strong generalization capability, requiring only a small amount of sample data for training to obtain a network with a certain accuracy. Therefore, BP neural networks are suitable for building a crab quality grading model.

### 4.2. GA-BPNN

From the mathematical viewpoint, the BP algorithm is a local search optimization method, which can approximate nonlinear mapping relations with arbitrary accuracy as long as hidden layers and nodes are sufficient and generalization capability is satisfactory. However, solving the global extremes of complex nonlinear functions with BP neural networks can be remarkably difficult because the initial weights and thresholds of BP are chosen randomly, thus easily falling into local extremes, which results in large prediction errors [28], and eventually leads to training failure. GA is a computational model that simulates the biological evolutionary process of natural selection and the genetic mechanism of Darwin’s theory of biological evolution. Compared with the inverse gradient descent method, GA can search multiple points in the solution space simultaneously without differential operations, which has superior robustness and a wide range of applications. The emergence of GAs has led to a new stage in the training of neural networks, and the use of GAs instead of BP algorithms to search the connection rights of neural networks is expected to solve the problem of BP networks falling into local minima [29].

**Figure 8:**
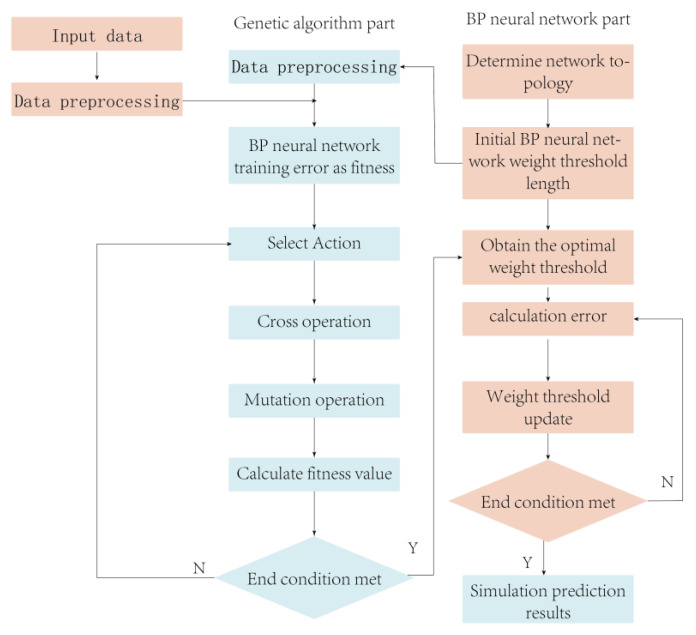
Genetic-algorithm-optimized BP process.

As shown in Figure 8, the advantages of both algorithms are utilized and GA is integrated with BP neural networks [30] to address the problems of slow computation, network instability, and generation of local extremes when neural networks are faced with nonlinear problems with complex mechanisms. The basic principle of optimizing BP neural networks using GA is to encode the neural network weights and thresholds using chromosomal properties of GA and then generate the initial population using a specific method while employing the classification accuracy error cost function of the BP neural network as genetic. The fitness function of the algorithm [31] uses genetics to manipulate individuals to select the best chromosomes as weights and thresholds for the neural network. The primary procedure for optimizing the BP neural network [32] using a genetic algorithm is as follows:

1. Randomly initialize the population: the crossover size, crossover probability, variance probability, and any set of weights are initialized and the connection weights of the neural network are encoded using binary, with the length of the binary encoding string depending on the choice of the initial number of populations.
2. Calculate the fitness value of the population and find the optimal individual from it: the binary code string of individuals is decoded, the fitness value of each individual in the current population is calculated and ranked using a fitness function, and network individuals are selected on the basis of a certain probability.

The reciprocal of the mean square error of the BP network is used in this paper as the fitness evaluation function *f* (*x*):

(6) $$f(x)=\frac{1}{\frac{1}{N}\sum_{j=1}^{n}\frac{{\left(Y-y\right)}^{{}^{2}}}{2}}$$

where *N* is the total number of training samples, *Y* is the network output, and *y* is the actual output of the sample.

(1) Select operation: Some individuals are selected as two parents for reproduction, and the roulette method is utilized for the selection operation. Individuals with high adaptation have a high inheritance probability to the next generation; conversely, individuals with low adaptation have a low inheritance probability to the next generation. The selection probability pi of each individual is as follows:
  (7) $$p_{j}=F_{j}/\sum_{j=1}^{e}F_{j}$$
  where *e* is the number of individuals in the population, and *F_j_* is the fitness value of individual *j*.

(2) Crossover operation: two paired individuals exchange some of their genes with crossover probability Pe, which, in turn, forms two new individuals using the real number crossover method, and the l1st and l2nd individuals are crossed at the *z*th gene by the following equation:
  (8) $$\begin{array}{l} h_{l1z}=h_{l1z}s+h_{l2z}\left(1-s\right)\\ h_{l2z}=h_{l2z}s+h_{l1z}\left(1-s\right) \end{array}$$
  where $h_{l1z}$, $h_{l2z}$ denote the genes of the l1st and l2nd individuals at the *z*th position, respectively, and *s* is a random number between [0, 1].

(3) Variation operation: For the variation operation, for each weight input position of the offspring chromosome, the variation operator randomly selects a value in the initial probability distribution with variation probability before performing the variation operation.

(9) $$h_{ij}=\left\{ \begin{array}{l} h_{ij}s+(h_{ij}-h_{\max})s_{1}(1-r/r_{\max}),r_{2}\geq 0.5;\\ h_{ij}s+(h_{\min}-h_{ij})s_{1}(1-r/r_{\max}),r_{2}<0.5; \end{array}\right.$$

In the Equation (9), the upper bound of the gene $h_{ij}$ is $h_{\max}$, the lower bound is $h_{\min}$, s is a random number located between [0, 1], $r_{2}$ is a random number, r is the current iteration number, and $r_{\max}$ is the maximum evolution number.

3. The new generation population is generated.
4. The completion of the algorithm is then determined. If a suitable individual is found, then the loop is skipped; otherwise, proceed to (2) to enter the next round of operation.
5. The best individual finally selected to obtain the connection weight coefficient of the neural network is decoded, and the sample is inputted for network training.

In this study, we employed MATLAB 2020b software to construct a three-layer BP neural network to investigate the relationship between inputs and outputs. The input layer consists of four neurons, whereas the output layer has a single neuron. The input parameters considered were crab weight, gender, fatness, and shell color. To prevent getting trapped in local optima, the genetic algorithm incorporates selection, crossover, and mutation operations. GA-BPNN randomly generates an initial population with N individuals, positions, and velocities. The optimal initial weights and thresholds obtained through the global optimization capability of GA are used as the initial weights and thresholds of the BP neural network. If the optimal fitness value meets the convergence criteria, the process is terminated. Otherwise, the process continues, and the individuals are subjected to selection, crossover, and mutation operations using the genetic algorithm to generate new individuals and populations. This process continues until optimal fitness meets the convergence criteria. The convergence criteria for the GA-BPNN network is set to 0.00001.

## 5. Experimental Validation

### 5.1. Equipment and Materials

**Figure 9:**
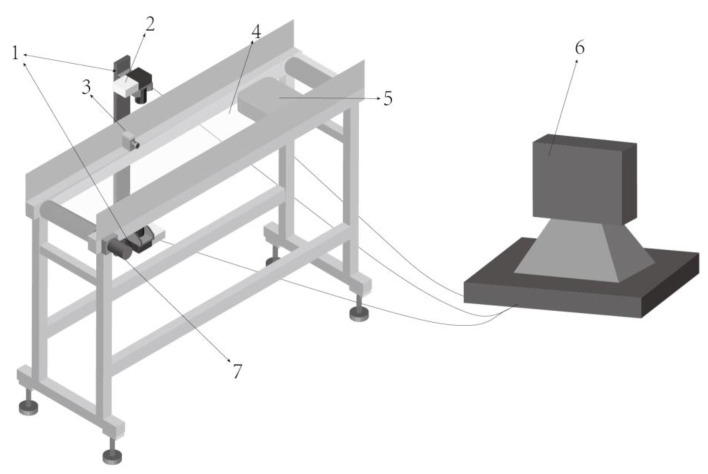
Schematic of experimental device. 1—CCD camera; 2—LED light; 3—photoelectric sensor; 4—transparent transmission belt; 5—load cell; 6—computer; 7—motor.

Abdominal and dorsal images of crabs must be obtained for quality analysis of crab images. Therefore, the structure of the device shown in Figure 9 is designed to complete the acquisition, processing, and feature extraction of the images.

The equipment models we chose are shown in Table 1, Table 2, Table 3 and Table 4.

**Figure 10:**
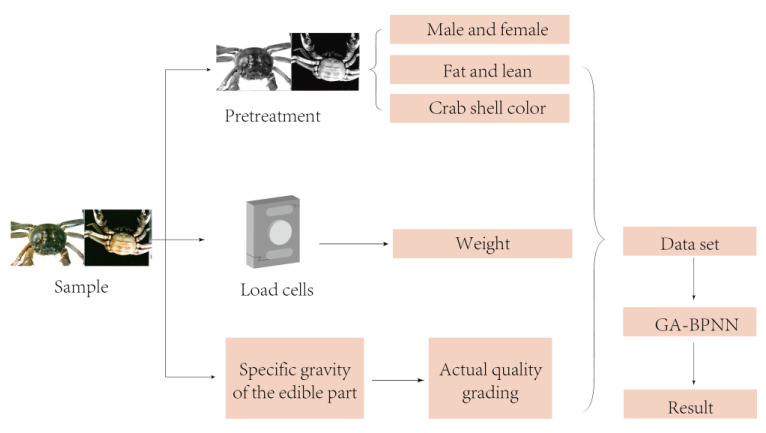
Process of crab quality detection based on image processing and GA-BPNN.

The experiment is completed in the above device. The grading process designed in this study is as follows: manually put the crabs on the transparent conveyor belt, and the transparent conveyor belt transports the crabs to the camera shooting area. Baffles are set up on both sides of the conveyor belt to ensure that the crabs cannot climb out. After the photoelectric sensor senses the arrival of the crab, it sends a signal to the CCD camera, and then the two CCD cameras installed above and below the conveyor belt take images of the crab’s abdomen and back, respectively, and send the images to the image processing unit to obtain characteristic information such as gender, fatness, and color of the crab shell. Then, the transparent conveyor belt continues to run to transport the crab to the working area of the load cell, and the weight information of the crab is obtained by the load cell. The gender, fatness, shell color, and weight information of crabs are used as input variables of the GA-BPNN model, and the model can judge and output the quality grade of crabs based on this information. The inspection procedure is shown specifically in Figure 10.

Eighty river crabs (40 male and 40 female crabs each) were selected as materials. Each crab was then numbered, and crabs placed on the conveyor belt were randomly selected. Afterward, images of the abdomen and back of the corresponding samples were collected under the same lighting environment, and the weight data measured by the load cell were recorded. Eighty samples were then prepared and photographed by continuously repeating the aforementioned step. Each crab was cooked and cut open after completion of image acquisition to disassemble the edible parts of crab paste, yolk, and meat, and the proportion of edible parts to the total weight of each sample was recorded; a large proportion indicates superior quality, which is a more accurate rating method than observing the quality with the naked eye. The four measured data were combined with the rating results to build a dataset [33,34,35] for subsequent use.

### 5.2. Testing GA-BPNN Model Performance

Determining the performance merit of the GA-improved BP neural network mainly observes its training capability and quality level prediction accuracy. Data training on the optimized BP neural network will be conducted to evaluate its training and prediction capabilities. Data sets are generally not all used as training sets to prevent overfitting. Data sets are divided before conducting data set training models. The data set is divided in this study into two parts: training and test sets. The training set is used to train the model using different training functions. The training set is used to train the model by using different training functions. The most common method for segmenting data sets is train_test_Split [36] of sklearn, which is convenient for dividing the data set into training and test sets. In this experiment, the selected data are randomly divided into a 7:3 ratio, with 70% for training sets and 30% for test sets. When conducting data training, instead of using randomly generated network weights, shortening the training time, and accelerating the convergence speed, employing the sample crab dataset for training using the optimal initial weights and thresholds obtained through the global optimization capability of GAs as the initial weights and thresholds of the BP neural network is necessary.

The total number of training sessions is set to 100, and the learning rate is generally taken as a number between 0 and 1. Meanwhile, the network initialization threshold is assigned to a random number in the interval (0, 1); after which, the learning rate is continuously adjusted through the neural network training to determine the amount of change in the weights generated in each training cycle. The learning rate is selected in the range of 0.01–0.8; a large learning rate may lead to system instability, whereas a small learning rate easily produces a long training time but may slow down the convergence speed. This condition can ensure that the error value of the network does not jump out of the error surface of the trough but can eventually converge to the minimum error value. Therefore, a small learning rate is generally used to ensure the stability of the system, and a learning rate of 0.01 is chosen for this experiment. Some other parameters set when performing data training are as follows: the minimum error of the training target is less than 0.000001, the display frequency is set to display data every 25 times, the momentum factor is 0.01, the minimum performance gradient is 1.00 × 10^−6^, and the maximum number of failures is set to 6. During the training process, the number of nodes in the hidden layer is continuously updated, the mean square error of the training set is determined, and the number of nodes corresponding to the smallest mean square error is selected as the final number of nodes in the hidden layer. The mean square error (*MSE*) is the most commonly used error calculation algorithm in machine learning algorithms. This algorithm is essentially the average value obtained from the square of the difference between the expected and actual outputs. The *MSE* is calculated as follows:

(10) $$MSE=\frac{1}{n}\sum_{i=1}^{n}w_{i}{\left(y_{i}-\hat{y_{i}}\right)}^{2}$$

In the formula: *n* refers to n pairs of input and output data, $y_{i}$ and $\hat{y_{i}}$, respectively, which refer to input and output data.

The mean square error corresponding to the number of nodes in different hidden layers is shown in Table 5.

**Figure 11:**
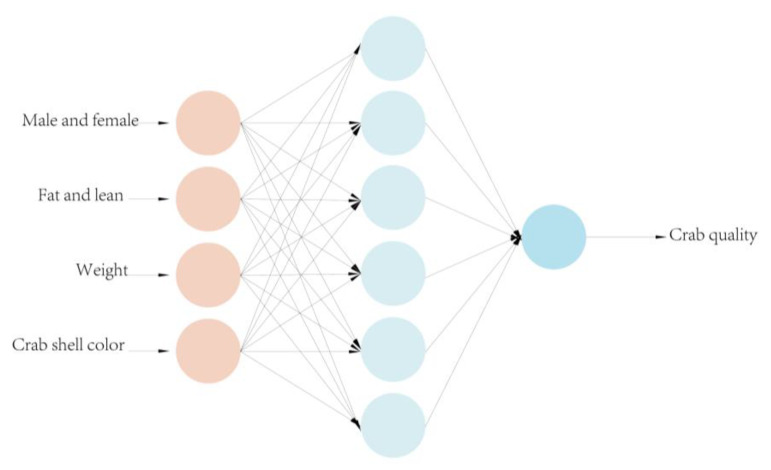
BPNN design structure.

The optimal number of nodes in the hidden layer is 6, and the corresponding mean square error is 0.0081613. Figure 11 shows the final structural model of the neural network after combining the input and output requirements.

**Figure 12:**
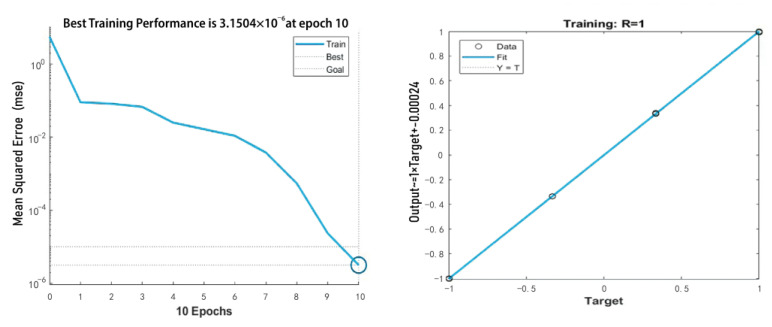
Training process.

Figure 12 shows that the mean square error reached the desired value and the linearity of fit met the requirements when the number of training sessions reached 10. Moreover, the system stabilized with a total error of 3.15 × 10^−6^ for the model output.

**Figure 13:**
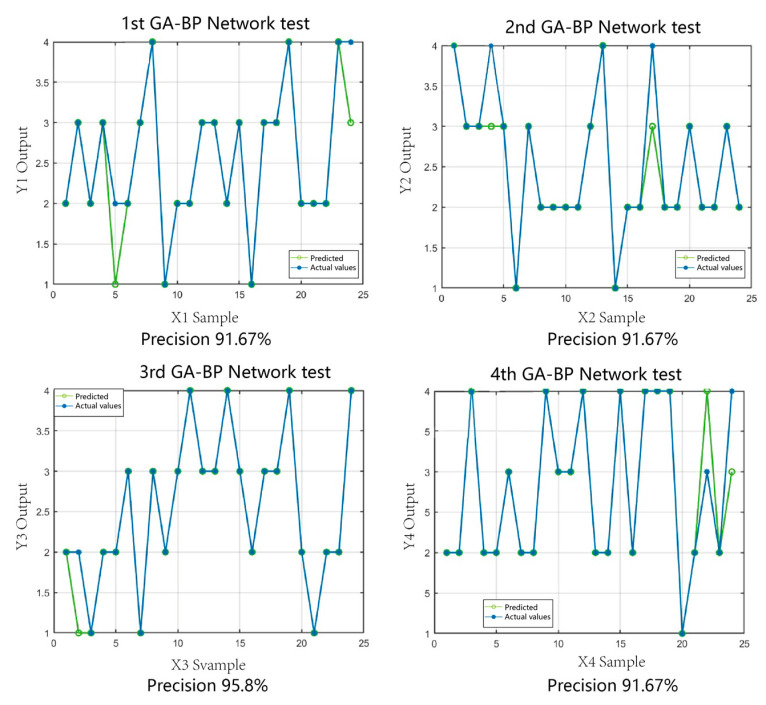
GA-BPNN prediction results.

A total of 24 sample crab data were randomly divided into the GA-BPNN model as the test set, while four random divisions were performed to ensure the accuracy of the results. The four sets of test results were then compared, and the model prediction accuracy was calculated. As shown in Figure 13, 22 sets of data met the expectation for the first time, and the grading accuracy reached 91.67%. Moreover, the second and fourth grading accuracies were both 91.67% and the third grading accuracy was 95.8%. The average of the four prediction accuracies was approximately 92.7%, which could meet the crab grading demand.

**Figure 14:**
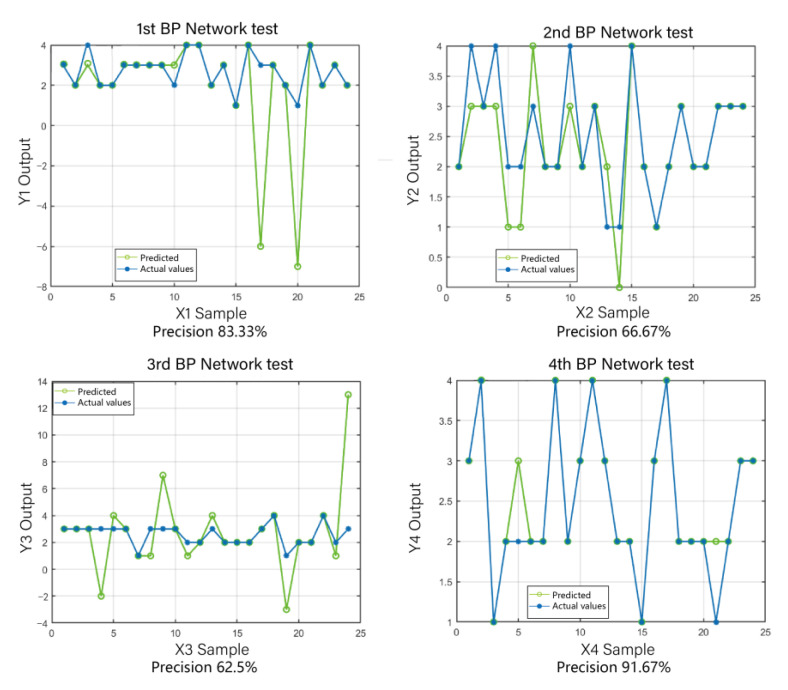
BP prediction results.

**Figure 15:**
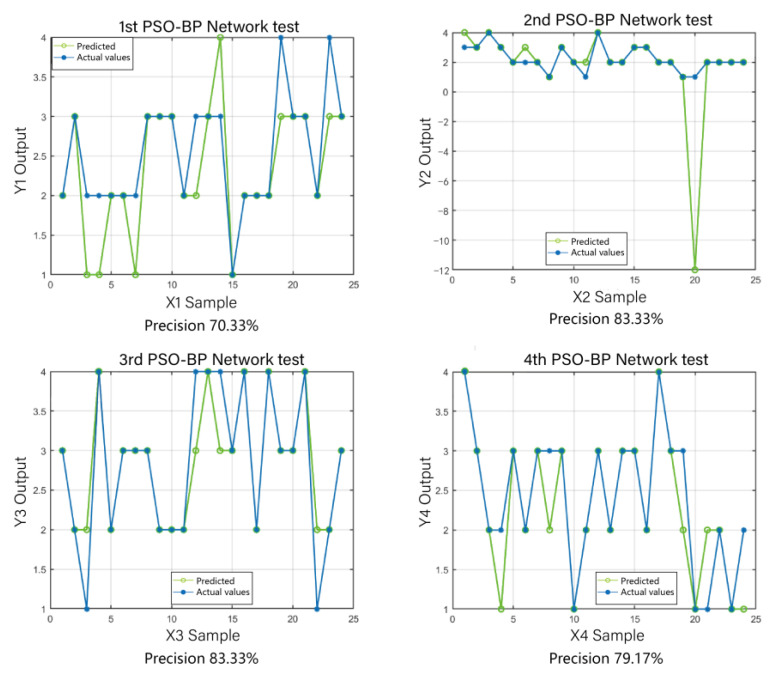
PSO-BP prediction results.

In this study, to compare GA-BPNN with other methods, we also employed the unoptimized BP neural network and the particle swarm optimization-based neural network (PSO-BP model) for crab classification. The particle swarm algorithm, as a traditional optimization algorithm, has been widely applied in various fields [37,38]. We conducted four tests for each of these two models and calculated their average prediction accuracies. From the results of the comparative experiments, it can be observed that the unoptimized BP neural network exhibited low overall prediction accuracy. Furthermore, different optimization methods had a significant impact on the prediction accuracy of the BP neural network. Among the three algorithm models, GA-BPNN produced prediction values that were closest to the actual values, and the optimization through the genetic algorithm significantly improved the model’s prediction accuracy. As shown in Figure 14, the prediction accuracies of the BP neural network algorithm were 83.33%, 66.67%, 62.5%, and 91.67%, with an average accuracy of only 76.04%. It is evident that the prediction accuracy of the BP algorithm is highly unstable, and the overall accuracy is significantly lower than that of GA-BPNN. From Figure 15, it can be observed that the prediction accuracies of the PSO-BP neural network algorithm were 70.83%, 83.33%, 83.33%, and 79.17%, with an average accuracy of 79.165%. Although there was an improvement compared to the BP algorithm, it is still far lower than the GA-BPNN model. Therefore, the utilization of genetic algorithm optimization for the BP neural network is highly necessary.

## 6. Conclusions

This paper applies machine vision technology to design a GA-BPNN model that can be utilized for the quality grading of river crabs. First, an experimental device was designed to collect images of the abdomen and back of the river crab, process the collected images, extract key features of the river crab, and input them into the GA-BPNN model for quality analysis. The main conclusions are as follows:

1. The commonly used algorithms for image processing were studied and analyzed, and the most suitable method was selected on the basis of the crab image characteristics. The weighted average and median filtering methods were used for image graying and noise reduction, respectively.
2. Four key features of river crabs, including gender, weight, fatness, and shell color, were selected for extraction. The method of feature extraction for river crabs was studied. The information on gender was obtained through shape matching and that on weight was obtained using a weighing sensor. The fatness was calculated by empirical Formula (5), and the carapace length was acquired through Hoff circle detection. The crab shell color was obtained by calculating the average gray value within the crab shell area.
3. GA was used to optimize the BPNN. The improved BP neural network based on GAs was employed to establish a multi-index classification model for river crabs against market standards. A dataset was established to train the model, the training and test sets were randomly divided four times, and the prediction effect of the optimized neural network was tested, demonstrating an average classification accuracy of 92.7%, which significantly outperformed the BP and PSO-BP models. The obtained findings indicate that the sorting accuracy of the crab quality sorting system based on machine vision and GA-BPNN can meet the sorting machine and market.

## Figures and Tables

**Table 1:** Camera parameters.

Name	Parameter
Model	InspectorP64x
Sensor	CMOS matrix sensor, gray value
Sensor resolution	1600 px × 1088 p × (1.7 Mpixel)
Camera lens	AFT-1214Mp telecentric lens
Lighting conditions	White ring LED light source

**Table 2:** Photoelectric sensor parameters.

Name	Parameter
Model	PM6-DUO5N
Sensing type	Diffuse reflection
Sensing distance	5 cm, White paper 10 × 10 cm
Emitting light	Infrared LED 940 nm
Operating voltage	DC12~24 V

**Table 3:** Motor parameters.

Name	Parameter
Model	4ik 25RA-C
Voltage	AC 220 V
Power	25 W
Current	0.25 A
Size	80 × 95 mm

**Table 4:** Load cell parameters.

Name		Parameter	
Range	0.5/1/2/510/20/50/100KN	Creep	±0.03% F.S/30 min
Nonlinear	0.03%	Impedance	350 ± 5 Ω
Sensitivity	1.0/2.0 ± 0.005% mV/V	Output impedance	350 ± 5 Ω

**Table 5:** Mean square error corresponding to the number of nodes.

Number of Hidden Layer Nodes	3	4	5	6	7
*MSE*	0.027966	0.05372	0.024212	0.0081613	0.032279

## Data Availability

The data presented in this study are available on demand from the first author at (xiaomaohua@njau.edu.cn).
